# Supplementary material for: MetaQTL: a package of new computational methods for the meta-analysis of QTL mapping experiments
Source: BMC Bioinformatics. 2007 Feb 8;8:49. doi: 10.1186/1471-2105-8-49 (PMC1808479; doi:10.1186/1471-2105-8-49)
Supplement: Additional File 7 — MetaQTL Package : jar file and tutorial. This Zip archive contains both the MetaQTL JAR file and the files of the tutorial. [file 1471-2105-8-49-S7.zip › org.inra.metaqtl/doc/org/metaqtl/numrec/NumericalUtilities.NRFunction.html]

NumericalUtilities.NRFunction


|  |  |  |
| --- | --- | --- |
| **Overview** \| **Package** \| **Class** \| **Use** \| **Tree** \| **Deprecated** \| **Index** \| **Help** | |  |
| **PREV CLASS**   NEXT CLASS | **FRAMES**    **NO FRAMES**     **All Classes** |  |
| SUMMARY: NESTED \| FIELD \| CONSTR \| METHOD | DETAIL: FIELD \| CONSTR \| METHOD |  |


---


## org.metaqtl.numrec Interface NumericalUtilities.NRFunction

**Enclosing class:**: NumericalUtilities

---

``` public static interface NumericalUtilities.NRFunction ```

**Author:**
:   jveyrier
    One dimensional function definition for Newton Raphson root
    finding algorithm.

---

| **Method Summary** | |
| --- | --- |
| `void` | `funcd(double x)`             Compute both the value and the derivate of the function in x. |
| `double` | `getDF()`             Returns the value of the derivate in x; |
| `double` | `getF()`             Returns the value of the function in x. |

**Method Detail**

### funcd

```
void funcd(double x)
```

:   Compute both the value and the derivate of the
    function in x.

    :   **Parameters:**: `x` -

---


### getF

```
double getF()
```

:   Returns the value of the function in x.

    :   **Returns:**

---


### getDF

```
double getDF()
```

:   Returns the value of the derivate in x;

    :   **Returns:**


---


|  |  |  |
| --- | --- | --- |
| **Overview** \| **Package** \| **Class** \| **Use** \| **Tree** \| **Deprecated** \| **Index** \| **Help** | |  |
| **PREV CLASS**   NEXT CLASS | **FRAMES**    **NO FRAMES**     **All Classes** |  |
| SUMMARY: NESTED \| FIELD \| CONSTR \| METHOD | DETAIL: FIELD \| CONSTR \| METHOD |  |


---
